# Activity Energy Expenditure Predicts Clinical Average Levels of Physical Activity in Older Population: Results from Salus in Apulia Study

**DOI:** 10.3390/s20164585

**Published:** 2020-08-15

**Authors:** Ilaria Bortone, Fabio Castellana, Luisa Lampignano, Roberta Zupo, Biagio Moretti, Gianluigi Giannelli, Francesco Panza, Rodolfo Sardone

**Affiliations:** 1 National Research Hospital, Population Health Unit—“Salus In Apulia Study”—National Institute of Gastroenterology “Saverio de Bellis”, Research Hospital, Castellana Grotte, 70013 Bari, Italy; castellanafabio@hotmail.it (F.C.); luisalampignano@gmail.com (L.L.); zuporoberta@gmail.com (R.Z.); f_panza@hotmail.com (F.P.); rodolfo.sardone@irccsdebellis.it (R.S.); 2 Orthopaedics and Trauma Unit, Department of Basic Medicine, Neuroscience, and Sense Organs, University of Bari Aldo Moro, 70124 Bari, Italy; biagio.moretti@uniba.it; 3 Scientific Direction, National Institute of Gastroenterology “Saverio de Bellis”, Research Hospital, Castellana Grotte, 70013 Bari, Italy; gianluigi.giannelli@irccsdebellis.it; 4 Neurodegenerative Disease Unit, Department of Basic Medicine, Neuroscience, and Sense Organs, University of Bari Aldo Moro, 70121 Bari, Italy

**Keywords:** actigraphy, self-report, measurement, energy expenditure, calibration, aging

## Abstract

Self-report questionnaires are a valuable method of physical activity measurement in public health research; however, accuracy is often lacking. Resolving the differences between self-reported and objectively measured physical activity is an important surveillance challenge currently facing population health experts. The present work aims at providing the relationship between activity energy expenditure estimated from wrist-worn accelerometers and intensity of self-reported physical activity (InCHIANTI structured interview questionnaire) in a sub-cohort of a population-based study on aging in Southern Italy. Linear regression was used to test the association between measured and reported physical activity. We found that activity energy expenditure predicted clinical average levels of PA assessed through InCHIANTI classification.

## 1. Introduction

Population aging along with the growing burden of chronic and multimorbidity brings with it an ever-increasing demand to improve disease prevention and align service delivery around the health and social care needs of the people. By 2050, one in six people worldwide will be over the age of 65 (16%), up from one in 11 in 2019 (9%). Limitation of mobility is a major public health problem along with physical inactivity [1] as: (1) they reduce the ability of individuals to move about their natural environment; (2) they inhibit the autonomous engagement of individuals in activities of daily living (ADL); (3) they decrease their capacity to interact socially; and (4) they have been found to be a primary risk factor for overall and coronary morbidity and mortality [2,3,4,5]. Multimorbidity, reported as having two or more chronic diseases [6], has been associated with decreased mobility and older age [7]

Given this scenario, it is essential to evaluate the modifiable factors that may help maintain independence in later life. Physical activity (PA) is commonly encouraged in order to reduce negative health effects. In fact, major organizations typically advocate daily physical activity for the general population, such as the World Health Organization recommendation who claimed for at least 150 min of moderate-intensity aerobic physical activity during the week or do at least 75 min of vigorous-intensity aerobic physical activity during the week or an equivalent combination of both [8]. Recent studies showed that limited physical function and older age are associated with levels of physical activity, and all three related to multimorbidity [9]. Higher PA levels could therefore prevent chronic diseases and mobility limitations and improve the quality and length of life in the older population [9]. Furthermore, there is considerable evidence linking physical activity to successful ageing in cross-sectional [10,11,12,13] and longitudinal studies [14,15]. Self-reporting methods, including questionnaires, have provided a powerful tool of PA assessment that can include useful and reliable knowledge on several health domains. However, evidence focusing on self-reported PA is vulnerable to prejudice coverage, and the recorded poor physical activity in older adults could be attributed to imprecise estimation [5,6,7].

Recent studies using objective measures of physical activity, such as accelerometry, have mostly examined direct associations of health determinants with total duration of physical activity. The determination of both time and magnitude spent in PA is significant in epidemiological research, since both are separate behaviors and risk factors. Menai and colleagues [16] showed that accelerometer data from a large sample of UK older adults suggest that successful agers practiced moderate to vigorous physical activity (MVPA) for longer duration and at higher intensity. Jaeschke and colleagues [13] investigated factors associated with habitual time spent in different activity intensities in the general German adult population (NAKO Study) using multiday 24 h-accelerometry. They showed that time spent in low-intensity activity was associated to both age and BMI with an inverse relationship: older adults spent more time in low-intensity while overweight–obese subjects less.

An important issue when dealing with the use of accelerometers to estimate physical activity is in the interpretation of the signals given by the devices, which need to be transduced into measurements with biological and/or behavioral meaning [17]. Recent evidence suggests that activity energy expenditure (AEE), defined as the component of total energy expenditure (TEE) that is caused by any kind of body movement produced by skeletal muscles (volitional exercise and non-exercise movements), has an important role in dictating lifespan [18,19]. In fact, previous studies have demonstrated that AEE declines through the 8th decade of life and is associated with age, lower walking speed at baseline and lean mass loss [20]. Questionnaires have historically been the main physical activity measurement instrument in epidemiological studies, although standard methods to validate different intensity of PA should be indirect calorimetry and doubly labeled water (DLW). Since these techniques may be not feasible in larger field and epidemiological studies, accelerometry has been introduced into the field of physical activity measurement, and several devices are currently in use in epidemiological studies, thus providing an objective output that might be used to estimate an individual’s AEE. However, there is still a lack of consistency of results in relating the self-reported physical status to specific energetic range. De Almeida Mendes et al. [21] carried on a systematic literature review to identify methodological features and findings from raw data calibration studies. They concluded that given the physical activity architecture that each approach assesses, linear regression, machine learning and cutting-edge methods posed compelling parameters of validity. Recently Metcalf and colleagues [22] demonstrated a calibration strategy to improve estimates of physical activity and sedentary behavior assessed through a global physical activity questionnaire (GPAQ). The InCHIANTI questionnaire [23] is a structured questionnaire specifically developed for the InCHIANTI (“Invecchiare in Chianti”, aging in the Chianti area), a population-based study where 1154 elderly persons living in two towns of the Chianti geographic area (Tuscany, Italy) have been cross-sectionally assessed in order to identify the factors contributing to the decline of mobility in late life [24]. Data from the InCHIANTI study have shown that physical activity and performance are correlated with lower systemic rates of proinflammatory biomarkers in older men and women. [25]; older adults who reported higher levels of physical activity in midlife had better mobility in old age with respect to those who were less physically active [26] and preserving or increasing PA levels may attenuate age-related decline in physical performance [27].

The present work aims at providing the association between activity energy expenditure estimated from wrist-worn accelerometers and different intensity levels of self-reported physical activity (InCHIANTI questionnaire) in a subset of a population-based study on aging.

## 2. Materials and Methods

### 2.1. Study Design and Population

Our research included individuals from the Salus in Apulia Study (previous Great Age Study), an ongoing population-based prospective cohort comprising of 2472 individuals aged 65 years old or older and residents in Castellana Grotte, a town located near Bari, Puglia, in the southeast of Italy. The Salus in Apulia Study (Great Age Study) focused on the sequence of lifestyle including diet, frailty, and other age-related impairments and age-related disease outcomes [28,29]. The Institutional Review Board of the National Institute of Gastroenterology “S. de Bellis” approved the Salus in Apulia Study with its measurements and data collections before starting in accordance with the Helsinki Declaration of 1975. All participants provided a written informed consent for the study.

This study used preliminary data of a sub-cohort of Salus in the Apulia Study of 50 old (+65) subjects, recruited consecutively from 2019 on a recall-based from the original cohort study. All potentially eligible patients were offered the opportunity to participate. Participants who accepted were fitted with the AX3 actigraph (Axivity, UK) during the last day of their “Salus in Apulia Study” clinic visit. The bioengineer at the Population Health Unit recruited eligible participants to wear the PA monitors. Those that used wheelchairs, or with impairments that prevented them from walking or wearing an accelerometer or who were cognitive impaired according to DSM V criteria were ineligible for PA monitors. The device, which contains a triaxial accelerometer with light and temperature sensors, was positioned on the dominant wrist. The sensor has been set to start recording from a specific date and time with a selected duration, in this case 7 days. Thus, participants were instructed to wear the AX3 for 7 consecutive days for 24 h. Before delivering the device, the bioengineer asked the participants whether the days they would wear the accelerometer were representative of their typical activity over that week, otherwise the device would have been delivered in a second moment. Participants with valid data, defined as daily wear time ≥16 h, for at least 5 valid days, were included in the analysis [30]. The study adhered to the “Standards for Reporting Diagnostic Accuracy Studies” (STARD) guidelines (http://www.stard-statement.org/) and the “Strengthening the Reporting of Observational Studies in Epidemiology” (STROBE) guidelines (https://www.strobe-statement.org/).

### 2.2. Accelerometer Variables

The actigraph collects movement in units of gravity (1 *g* = 9.81 m s^−2^) at a sampling rate of 100 Hz per second by a 12 bit analogue-to-digital converter (dynamic range, ±8 *g*). After the collection period, participants returned the AX3 to the Research Unit. Data were processed in R using the GGIR package (v. 2.0-0, cran.r-project.org/web/packages/GGIR/index.html) [31]. Calibration error was estimated based on static periods in the data and corrected if necessary [32]. Non-wear time was estimated on the basis of the standard deviation and the value range of the raw data from each accelerometer axis: a block was classified as the non-wear time if the standard deviation of the 60 min window was less than 13.0 m*g* [33]. Only the activity data between 5:00 a.m. and 11:00 p.m. (deemed as the waking period) were considered. Sleep periods were detected using a previously validated algorithm, the heuristic algorithm HDCZA for SPT-window detection [34].

The Euclidean norm minus one (ENMO) was used to quantify the acceleration associated with the movement recorded and represented in milligravity, m*g* [33]. Negative values have been rounded to zero and ENMO values have averaged over 5 s epochs. We applied the proposed cut-points for adults by Hildebrand and colleagues (2014, 2017) to estimate time spent in different acceleration levels [35,36] and defined as:

- Sleep or SIB (the periods of time during which the z-angle does not change by more than 5 degrees for at least 5 min);
- Other inactivity (threshold under 30 m*g*);
- Light (ENMO threshold over 30 m*g*);
- Moderate (ENMO threshold over 100 m*g*);
- Vigorous (ENMO threshold over 400 m*g*).

We then applied the model reported in White et al. [37,38] to predict the energy expenditure related to physical activity (AEE) and expressed in J min^−1^ kg^−1^ (or kJ day^−1^ kg^−1^). Finally, the sum of predicted activity energy expenditure and predicted resting energy expenditure (REE) from the simplest model (using only age, sex, height and weight) [39] represented the expected total energy expenditure (in MJ day^−1^) after dividing the result by 0.9 to compensate for diet-induced thermogenesis [40]. For physical activity to be categorized as MVPA, 5s-epoch mean acceleration (ENMO) at or above 100 m*g* was needed. In order to minimize signals related to unintended wrist movement, we only preserved activities lasting at least 10 min for which 80% of the activity met the 100 m*g* threshold criteria (bouts).

Finally, we have classified each participant according to WHO PA recommendations, which indicates bouts of ≥10 min as time spent in moderate or vigorous activity [8]. We also determined the cumulative time spent in 10-min bouts in moderate or very vigorous (VV) activity, divided the sum by the number of assessment days, and multiplied by 7 to produce average week estimates. “Meeting WHO recommendation” was defined as having achieved at least 150 min of estimated physical activity throughout the week or at least 75 min of VV physical activity throughout the week. Otherwise, we calculated if the participants had obtained a “metabolic equivalent”, as stated, though not described, by the WHO. The accomplishment of the WHO criteria is equal to achieving 450 METs (metabolic equivalent tasks) bouts per week by using 3 and 6 METs as a threshold for moderate and vigorous activity, respectively [8]. In order to consider higher activity intensities at both strength rates, we multiplied the averages of weekly bouts in moderate and VV activity by 4 and 8 METs, respectively [41]. Participants achieving at least 450 METs bouts per week based on moderate and VV activity were categorized as “meeting WHO recommendation”, whereas participants not fulfilling any of the aforementioned requirements were classified as “not meeting WHO recommendation”.

### 2.3. Assessment of Physical Activity

Physical activity was assessed using a questionnaire administered to the interviewer [25,26]. Subjects were requested to show their average amount of physical activity throughout the past year. One of the following six response categories that involves length, frequency, and strength of physical activity may be selected for each age group: (0) no physical activity (or bedridden), (1) minimal physical activity, (2) light physical activity consisting of 2–4 h per week not accompanied by sweating (e.g., walking), (3) moderate physical activity performed 1 to 2 h per week accompanied by sweating or light physical activity not accompanied by sweating for >4 h per week, (4) moderate physical activity carried out for more than 3 h per week accompanied by sweating, and (5) physical exercise attended regularly that required maximal strength and endurance several times per week.

Furthermore, physical activity scale for the elderly (PASE) was used to evaluate participants’ usual physical activity since it has been recognized as a validated instrument for the assessment of physical activity in epidemiologic studies involving persons 65 years and older [42]. The PASE scale considers PA performed over a week expressed as participation in leisure activities, sport, and recreation and categorized according to frequency and duration. Activities mostly related to a sitting position, like paid or unpaid work, were recorded in total hours per week. A dichotomous variable (“yes” or “no”) was checked for housework (light and heavy), lawn work/yard care, home repair, outdoor gardening, and caring for other people. The total PASE score was determined as the sum of all activities after multiplying the amount of time spent in each activity (hours/week) or participation (yes/no) in an activity by the empirically derived item weights. After stratification in terciles, a PASE score below 40 indicated sedentary behaviors; in the range from 41 to 90 documented light physical activity and over 90 was indicative of moderate to intense activity [43].

### 2.4. Covariates

Age, sex, smoking history, and education were self-reported using a standardized interview questionnaire. Plasma glucose was determined using the glucose oxidase method (Sclavus, Siena, Italy), while the concentrations of plasma lipids (triglycerides and total cholesterol) were quantified by automated colorimetric method (Hitachi; Boehringer Mannheim, Mannheim, Germany). Hemoglobin, insulin, alanine aminotransferase (ALT/GPT) aspartate transaminase (AST/GOT) and Gamma glutamyl transferase (GGt) were measured using automatic enzyme procedures. Global cognitive function was assessed using the Italian version of the Mini-Mental State Examination (MMSE). Body mass index was calculated as the weight in kilograms divided by height in meters squared. Depressive symptoms were evaluated using geriatric depression scale (GDS).

### 2.5. Statistical Analysis

We performed statistical analysis of baseline variables as the median and range for continuous variables and proportion (%) for the frequency of categorical variables (Table 1). The whole sample (*N* = 50) was divided in three groups according to the InCHIANTI classification levels. The normality of distribution was assessed for each variable using the Shapiro’s test. Kruskal Wallis sum rank test was performed based on methodological suitability, to assess differences of collected variables between groups (Table 2 and Table 3). A Spearman’s correlation matrix was built for all continuous variables in order to assess the presence of significant correlations (data not shown). *p*-values less than or equal to 0.05 were considered statistically significant, with 95% confidence intervals.

Two linear regression models were built on physical activity energy expenditure, AEE (kJ day^−1^ kg^−1^), as a dependent variable and InCHIANTI levels as a regressor as follows: (1) partially adjusted model including age, sex and BMI as major confounders and (2) fully adjusted model by including also MMSE and GDS (Table 4). The selection of confounders was made by considering both the univariate significance with AEE and major covariates such as age, BMI and gender.

**Figure 1:**
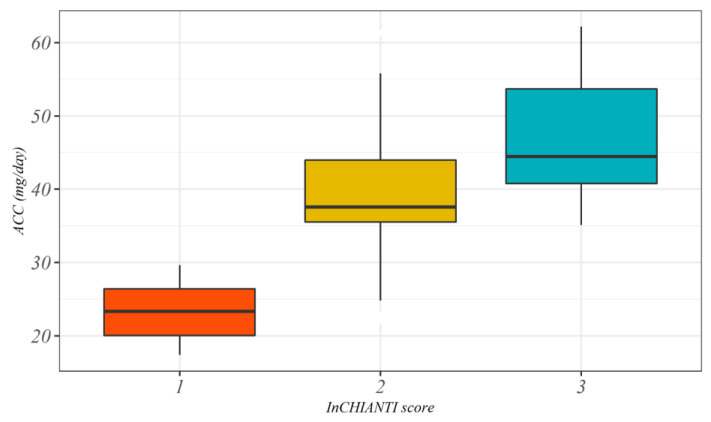
Box plot of mean acceleration per day estimated from actigraph for the three declared levels of PA according to the InCHIANTI questionnaire (the three groups significantly different from each other, *p* < 0.01).

**Figure 2:**
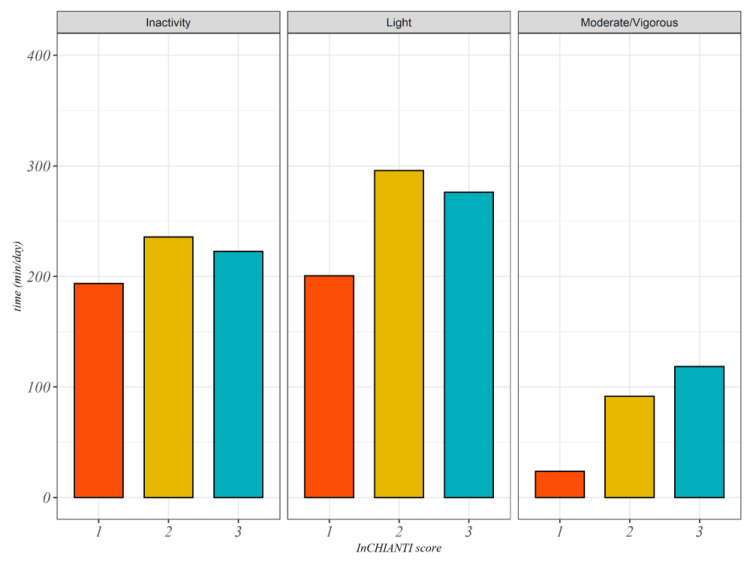
Box plot of mean acceleration per day and activity energy expenditure estimated from actigraph for the three declared levels of PA according to InCHIANTI questionnaire.

Figure 1 describes the distribution of AEE divided by InCHIANTI classification score as Box Plots. Figure 2 shows the time spent in different intensity of physical activity divided by InCHIANTI levels classification.

Statistical analyses were performed using RStudio software, Version 1.2.5042 (RStudio, Inc., Boston, MA, USA).

## 3. Results

Descriptive statistics are presented for anthropometric, lifestyle characteristics, and cognitive status in Table 1. Most participants provided 24 h-accelerometry data for 7 observation days (78%; 5 days: 8%; 6 days: 14%; Table 1). Additionally, median (interquartile range (IQR)) for energy expenditure and minutes of inactivity, light and moderate to vigorous PA are presented in Table 2. A total of 50 subjects aged 76.63 ± 5.97 returned the device after one week. The cohort was equally distributed by age and gender (27 males, mean age = 77.12 ± 6.28; 23 females, mean age = 76.05 ± 5.53) and no differences emerged in terms of sociodemographic, cognitive or emotional status. According to actigraphy measurements, median predicted energy expenditure (TEE) was 10.55 (8.11–13.86) MJ day^−1^ for men and 9.92 (7.87–12.10) MJ day^−1^ for women, of which 5.48 (4.76–6.73) MJ day^−1^ and 5.09 (4.71–6.07) MJ day^−1^ was resting energy expenditure (REE) respectively for men and women. While TEE was not significantly different according to gender (*p* = 0.37), REE was significantly higher in men than women (*p* = 0.01). Furthermore, men spent more time in low-intensity activity than women (*p* = 0.07) but no significant difference has been found in time in inactivity.

Median (range) activity-related acceleration (ACC) per day was 37.25 m*g* (17.37–62.18) on the dominant wrist and did not differ between gender (respectively 35.45 m*g* (17.37–61.33) for male and 38.16 m*g* (18.76–62.18) for female, (*p* = 0.15). Less than one third of participants met the WHO PA recommendation, with 12% fulfilling the criteria of 150 min/week moderate activity spent in bouts of at least 10 min, or the metabolic equivalent of 450 METs bouts per week.

Total bouts of at least 10 min per week spent in MVPA were negatively correlated to age, as METs bouts per week (r_s_ = −0.3, *p* < 0.05), thus indicating a decrease of moderate to vigorous physical activity while getting older. Self-reported physical activity measured with PASE was moderately negatively correlated with both age and BMI (r_s_ < −0.4, *p* < 0.01): older and overweight subjects reported a more sedentary lifestyle (low PASE score). Cognitive status has been found negatively correlated with BMI, resting and total energy expenditure (REE, TEE): subjects who scored less than 26 for MMSE were both physically inactive according to the WHO PA recommendation and overweight (BMI > 25 kg m^−2^). The intensity of physical activity (ACC), interpreted as the magnitude of acceleration the device was subjected to at each measurement, was related to all the PA variables in terms of time, energy expenditure (r_s_ > 0.8, *p* < 0.001) and amount of METs bouts per week (r_s_ = 0.7, *p* < 0.001). Additionally, ACC was moderately negatively correlated to age (r_s_ < −0.4, *p* < 0.05) increased age was therefore associated with a decrease in the magnitude of acceleration measured by the actigraph during the day.

In Table 2 we reported the description of the whole sample divided according to the InCHIANTI level: low physical activity corresponded to older subjects with lower mean acceleration per day (*p* < 0.001) and lower mean activity energy expenditure (*p* < 0.001). Furthermore, subjects who declared light levels of physical activity were significantly different from the other groups in terms of non-wear time. No significant difference emerged neither in sociodemographic variables nor for cognitive or emotional status.

Figure 1 shows the distribution of mean physical activity expressed as milligravity per day for the three levels of InCHIANTI reported by the participants. There was a clear correspondence between the mean amount of acceleration and the different classes of the InCHIANTI questionnaire: subjects with low mean acceleration per day have self-reported lower levels of PA, corresponding to the first class of InCHIANTI, while more active subjects showed increased mean values of PA (cut-off around 30 m*g*). Furthermore, subjects who declared moderate intensity of PA were significantly different from subjects who reported light PA (*p* < 0.05).

In Table 3 we reported other variables, energy expenditure (resting metabolic rate—REE, activity energy expenditure—AEE and total energy expenditure—TEE, expressed as MJ per day), Time spent in different intensities of PA and meeting WHO recommendation, for all the declared InCHIANTI levels of physical activity. There was a correspondence among the estimated time spent in light and moderate to vigorous physical activity and the declared InCHIANTI levels, in particular subjects characterized by low physical activity spent significantly less time in moderate to vigorous PA with respect to other categories. In addition, they were characterized by lower values of energy expenditure, even though no differences were found among subjects who declared light or moderate PA according to the InCHIANTI classification.

In Figure 2 we showed the distribution of time spent in different intensities of physical activity measured through actigraphy according to the InCHIANTI classification. Participants who declared to be more active (InCHIANTI 3) spent more time in moderate to vigorous physical activity than other groups, although the time spent in inactivity and light activities was higher. No statistically significant difference emerged in terms of time spent in inactivity and for the subjects who declared light and moderate PA according to the InCHIANTI questionnaire in terms of the time spent in light PA.

Finally, we carried out two linear regression models in order to evaluate whether the activity energy expenditure measured through a wrist-worn accelerometer was able to predict the different levels of the InCHIANTI structured interview questionnaire (Table 4). In the partially adjusted model examining the association between self-reported PA intensity and physical activity energy expenditure measured through actigraph, AEE, the association with increasing levels of self-reported PA according to the InCHIANTI questionnaire was higher (*p* < 0.01 for all classes). Female gender was significantly associated to AEE (*p* < 0.05), while no other variables contributed significantly to the models. In the fully adjusted model, the association between the InCHIANTI classification of physical activity and activity-related energy expenditure persisted and female gender lost its significance. For a unit change in the InCHIANTI score from 1 to 2, the activity-related energy expenditure increased 17,434 kJ min^−1^ day^−1^ for male and 24,576 kJ min^−1^ day^−1^ for female. For a unit change in the InCHIANTI score from 2 to 3, the AEE increased 23,449 kJ min^−1^ day^−1^ for male and 30.591 kJ min^−1^ day^−1^ for female. The age, as the BMI, seems to have no effect on the model (*p* > 0.05), even though the direction of the association indicated that older/obese subjects would have lower levels of activity-estimated energy expenditure. Additionally, cognitive or emotional status showed no significant effect on the models (*p* > 0.05). However, for each unit increase of the MMSE score, the activity-estimated EE would increase by 0.093 kJ min^−1^ day^−1^, while an inverse relationship has been observed for the GDS score: for each unit change in GDS, the energy expenditure related to PA (AEE) decreased by −0.437 kJ min^−1^ day^−1^. However, almost 25% of the subjects did not have the score for both cognitive and emotional status, possibly limiting the effects of these variables on the model.

## 4. Discussion

Physical inactivity is a key risk factor for total and cardiovascular morbidity and mortality [5] and unfortunately a large proportion of the population worldwide is insufficiently physically active [1]. The present study aimed at providing the association between activity-related energy expenditure (AEE) estimated from wrist-worn accelerometers and different intensity levels of self-reported physical activity (InCHIANTI structured interview questionnaire) in a subset of a population-based study on aging. Although standard methods to validate different intensity of PA should be indirect calorimetry and doubly labeled water (DLW), these techniques may be not feasible in larger field and epidemiological studies. Therefore, questionnaires have historically been the main physical activity measurement instrument in epidemiological studies. However, evidence suggests that the reported low physical activity of older adults may be the effect of imprecise measurement [5,6,7]. On the contrary, accelerometers are currently a feasible alternative to objectively measure physical activity [44] and various activity monitors are gaining more widespread use for providing objective measures of physical activity [45,46]. We then recruited eligible participants from our population-based study, Salus in Apulia, to wear a validated wrist-worn accelerometer-based monitor for 7 days [37] and to effectively measure the intensity of physical activity self-reported through a structured interview questionnaire, InCHIANTI. Our results showed that less than one third of participants met the WHO PA recommendation, with 12% fulfilling the criteria of 150 min/week moderate activity, respectively, spent in bouts of at least 10 min, and 12% meeting the metabolic equivalent. The results showed that older subjects who declared lower levels of PA (InCHIANTI class 1) spent more time in light activities than subjects belonging to higher classes and the amount of energy expenditure related to PA was lower than other groups, thus confirming the reported score. Changes in activity intensities with age are reasonable due to age-related decrease in cardio-respiratory and musculoskeletal health, thereby restricting resources for extremely intensity PA output. Furthermore, psychosocial barriers such as reduced social support for PA or feeling “too old” for high-intensity PA may contribute to the significant decrease in moderate-to-vigorous activity and the simultaneous increase in low-intensity activity with higher age [47]. Interestingly, age was not associated to time inactivity, suggesting that while higher intense activity may diminish with age, accelerometer-based inactivity time may not differ significantly. These findings were consistent with previous investigations from cross-sectional and longitudinal studies that reported higher age related to less time in VV activity and more time in low-intensity activity, including 24 h-accelerometry-based data [13,41]. Although age was related to the intensity of PA (ACC) and to activity-related energy expenditure (AEE in kJ day^−1^ kg^−1^), we did not observe an influence of age on the linear regression model. This effect could be probably due to the fact that the sample was limited, and so the adjustment for the InCHIANTI classification might have reduced the strength of the previously observed correlation. However, the direction of the relationship is negative, thus indicating that being older was associated with lower physical activity-related energy expenditure.

We also observed an inverse moderate correlation between cognitive status and body mass index, resting and total energy expenditure. It should be noticed that the basic metabolic rate (REE) has been estimated through a simplest model using only age, sex, height, and weight [39] so the major contribution to the observed relationship was due to BMI. Previous studies have examined the association between BMI over the adult life course and cognition in late midlife and they found that subjects with a higher BMI in midlife were at higher risk of cognitive impairment [48,49]. Additionally, a slight correlation has also been observed between BMI and time spent in inactivity. This effect can possibly be partially clarified by the fact that BMI is strongly associated with body weight and so that overweight/obese persons may more likely be less active (in terms of acceleration) due to the higher effort of movements; in contrast, the energy expenditure related to PA does not necessarily differ from persons with lower BMI [40]. Finally, we have shown that mean activity-related energy expenditure in this population was greater in women than men. It remains to be investigated whether these results indicate true differences in physical activity between sexes or are a function of the gender difference in the relationship between wrist acceleration and true activity.

The calibration of questionnaires related to PA to objectively measure physical activity is a useful way to remove noise from self-reported data. In fact, this approach allows researchers to correlate self-reported PA results with quantitative indicators as well as to help explain how these two methods could be applied together. In the present study, we demonstrated the effectiveness of the described approach by calibrating the InCHIANTI structured interview questionnaire [23] to 7 days of accelerometer-based data. The future application of the proposed calibration model involves enhanced PA estimates in broad sample surveillance results, experimental and pre-/post-measurement studies where accurate PA measurements are not conceivable. Our database currently includes 2472 subjects assessed through the InCHIANTI structured questionnaire. We will be then able to provide for a clearer understanding of PA determinants, as well as the dynamic interaction between PA and health effects in our community. As previously reported, using multiday 24 h-accelerometry to assess habitual PA in a population-based study highlighted distinct associations with biological, behavioral, socioeconomic, and sociocultural factors [13], thus setting clear objectives for public health interventions aiming to increase activity.

The low-to-medium magnitude of the relationship between physical activity and multiple health effects may be strengthened if the measuring methods of physical activity show high specificity and consistency, especially at the person level [50]. It is essential as we strive to assess the physical activity of particular subjects, like the frail population in clinical practice. Recently, Wanigatunga et al. [51] proposed that highly irregular habits of physical exercise might be an early indication of reduced ability culminating in premature mortality. To this end, recording the fragmentation of physical activity is a critical phenotypic predictor for the degradation in free-living patterns in physical activity correlated with mortality. Furthermore, developments in the comprehensive PA evaluation offer an ability to fully appreciate age-related adjustments in the magnitude, period, and pace of daily activities and the resulting impacts on wellbeing and functional status. Schrack and colleagues demonstrated that time spent in moderate or higher-intensity activities will not be lower with age after examining improvements in metabolism, functional capacity, and subclinical disease burdens [52].

### Strengths and Limitations

Strengths of our study include the focus on multi day triaxial 24 h-accelerometry to assess PA in a sample of older adults without functional decline and major disease, thus allowing for a more detailed and unbiased PA measurement than in previous studies utilizing self-reported PA or uniaxial accelerometry. Additionally, we included a validated PA questionnaire in population-based settings, which increases its usability for other researchers.

Limitations of this study include the analytic sample being rural Mediterranean population, physical activity profiles measured at 1 time point, a sample of older adults who tend to be higher functioning than the general older adult population. Furthermore, although participants were asked to follow their daily routine, the involvement in the study and the use of actigraphy may affect PA behavior. However, in previous studies, no evidence of behavioral bias regarding PA when using a wrist-worn accelerometer has been observed [53]. A further limitation may be that accelerometer data does not entail the domain of PA, such as leisure-time or occupational PA. However, for activity intensity determination, we considered only the activity data between 5:00 a.m. and 11:00 p.m. (deemed as the waking period). The small sample could also have potentially limited the range of PA observed to be smaller than that, which might be observed in a larger study. Finally, the fact that our sample reported an InCHIANTI score from 1 to 3 limited the possibility to explore the association with higher levels of intensity of physical activity (moderate to vigorous). However, it should be noticed that in the older population the first three categories were the most common reported: in Patel and colleagues (2007) most of the enrolled subjects (74.7%) belonged to those categories [26].

## 5. Conclusions

Physical activity is important as an exposure and outcome factor for research in the healthcare sector. Although accelerometer-based assessment of PA is widely used in public health research, self-reporting methods, including questionnaires, remain powerful instruments of PA evaluation that can offer specific and useful details on domains in addition to PA volume estimates. Furthermore, PA self-reporting methods require a review of historical results where quantitative indicators have not been applied in all assessment times. To our knowledge, this is the first demonstration of the validity of estimated activity energy expenditure from wrist-worn accelerometers for average physical activity intensity assessed through InCHIANTI structured interview questionnaire. Although estimated activity EE has been approximated by using regression formulas, which may lead to larger prediction errors when using these equations for longer and free-living studies, our study shows a novel approach to calibrate and equate self-reported InCHIANTI results with quantitative PA indicators. Furthermore, it allows researchers to harmonize various measurements of the same indicator and to consider how self-reported and objective variables should be used together to build a more consistent PA calculation. Finally, improved PA measurement will contribute to a clearer definition of PA factors and the dynamic interaction between PA and health effects as the need for further age-specific PA studies to support potential strategies and public health recommendations.

## Figures and Tables

**Table 1:** Description of sociodemographic variables of the whole sample (50).

	Median	Range
AGE (Years)	76	66 to 90
GENDER (Female)	23 (46.0)	--
BMI (kg m^−2^)	28.04	22.59 to 40.54
SMOKING (yes)	2 (4.8)	--
EDUCATION (Years)	5.5	3 to 17
MMSE	27.4	21.9 to 30
GDS	1.5	0 to 24
PASE	88.33	2.86 to 311
InCHIANTI		
*1*	13 (26.0)	--
*2*	26 (52.0)	--
*3*	11 (22.0)	--
*4*	--	--
*5*	--	--
Meeting WHO recommendations (Yes)	6 (12.0)	--

All data are shown as median and IQR (interquartile range) for continuous variables and as (%) for proportions.

**Table 2:** Description of sociodemographic variables, metabolic biomarkers, neuropsychological status and general accelerometer-based PA parameters according to the InCHIANTI classification.

	Physical Activity Intensity (InCHIANTI)			*p*-Value
	1	2	3	
AGE (Years)	80 (68 to 89)	77.5 (71 to 90)	71 (66 to 87)	**0.01** *
GENDER (Female)	46.2 (6)	13 (50.0)	4 (36.4)	0.75 ^a^
SMOKING (Yes)	--	1 (3.8)	1 (16.7)	0.32 ^a^
EDUCATION (Years)	5 (5 to 16)	7.5 (3 to 17)	6.5 (5 to 13)	0.81 *
BMI (kg m^-2^)	29.4 (24.0 to 32.7)	28.1 (23.1 to 40.5)	25.7 (22.6 to 34.4)	0.44 *
FFM (kg)	49.2 (42.4 to 65.6)	45.4 (35.4 to 66.9)	52.4 (39.8 to 66.6)	0.28 *
PA (°)	4.65 (3.6 to 6.6)	5.4 (4.4 to 6.7)	7.2 (3.7 to 12.0)	**0.04** *
SMI (kg m^−2^)	9.3 (6.6 to 12.2)	8.5 (5.2 to 12.0)	9.15 (6.4 to 13.1)	0.6 *
Hemoglobin (g dl^−1^)	14.05 (13 to 16.8)	13.75 (8.1 to 16.5)	13.5 (12.5 to 16.9)	0.74 *
FBG (mg dl^-1^)	93 (86 to 162)	93.5 (77 to 146)	93.5 (87 to 102)	0.86 *
GOT (U/L^-1^)	20 (17 to 33)	20.5 (16 to 55)	21 (18 to 23)	0.96 *
GPT (U/L^-1^)	17.5 (10 to 23)	17 (11 to 46)	17 (16 to 20)	0.93 *
GGt (U/L^−1^)	12.5 (9 to 22)	17.5 (10 to 53)	24 (9 to 80)	0.05 *
Cholesterol (mg dl^-1^)	167.5 (123 to 264)	175 (106 to 237)	178.5 (149 to 218)	0.93 *
Triglycerides (mg dl^-1^)	92 (50 to 210)	67.5 (32 to 176)	107 (80 to 133)	**0.03** *
Insulin (UI)	7.65 (2.8 to 11.5)	6.85 (2.40 to 17.0)	6.32 (4.50 to 13.70)	0.91 *
MMSE	27.6 (21.9 to 30)	27.4 (22.3 to 30.0)	27 (26.9 to 30.0)	0.87 *
GDS	5 (0 to 12)	1 (0 to 24)	1 (0 to 13)	0.26 *
PASE	82.6 (2.9 to 106.7)	95.3 (27.6 to 311.0)	92.9 (65.6 to 115.1)	0.16 *
Valid days	7 (5 to 7)	7 (5 to 7)	7 (5 to 7)	0.88 *
Non wear hours	0.04 (0 to 0.35)	0 (0 to 0.20)	0 (0 to 0.04)	**0.04** *
ACC (m*g* day^-1^)	23.3 (17.4 to 29.6)	37.5 (22.1 to 61.3)	44.4 (35.1 to 62.2)	**<0.001** *
AEE (kJ day^-1^ kg^−1^)	38.0 (30.7 to 45.7)	55.4 (36.5 to 84.6)	63.9 (52.5 to 85.6)	**<0.001** *

* Wilcoxon sum rank test; ^a^ Fisher’s exact test.

**Table 3:** Descriptive statistics of accelerometer-based PA variables in the InCHIANTI classification.

	Physical Activity Intensity (InCHIANTI)			*p*-Value
	1	2	3	
Energy Expenditure (MJ day^−1^)				
REE	5.37 (4.76 to 6.44)	5.31 (4.71 to 6.73)	5.51 (5.03 to 6.32)	0.67 *
AEE	2.53 (2.15 to 3.51)	3.96 (2.92 to 6.51)	4.39 (4.00 to 5.22)	**<0.001** *
TEE	8.83 (7.87 to 10.97)	10.24 (8.605 to 13.86)	10.91 (10.50 to 11.89)	**<0.001** *
Time (min day^−1^)				
Inactivity	193.6 (105.4 to 311.3)	235.75 (106.1 to 315.9)	222.5 (180.60 to 377.80)	0.48 *
Light	200.4 (108.6 to 249.2)	295.9 (142.5 to 397.8)	276.2 (217.2 to 399.90)	**<0.001** *
Moderate to Vigorous	23.65 (10.82 to 69.62)	91.63 (36.93 to 226.57)	118.37 (83.24 to 189.21)	**<0.001** *
WHO PA Recommendation				
meeting (Yes)	--	1 (3.8)	5 (45.5)	**0.001 ^a^** *

* Wilcoxon sum rank test; ^a^ Fisher’s exact test.

**Table 4:** Linear regression model on activity energy expenditure (AEE; kJ day^−1^ kg^−1^) as dependent variables and regressors.

		β	Standard Error	CI 95%	*p* Value
Partially Adjusted	*(Intercept)*	51.55	25.46	1.66 to 101.44	**0.04**
	InCHIANTI [2]	19.96	3.47	13.17 to 26.76	<**0.01**
	InCHIANTI (3]	27.29	5.16	17.18 to 37.41	<**0.01**
	AGE (years)	−0.13	0.28	−0.68 to 0.43	0.65
	GENDER (Female)	6.42	3.03	0.48 to 12.36	**0.03**
	BMI (kg m^−2^)	−0.26	0.37	−0.98 to 0.46	0.48
Fully Adjusted	*(Intercept)*	79.44	39.374	2.269 to 156.61	**0.04**
	InCHIANTI [2]	17.434	4.192	9.218 to 25.65	<**0.01**
	InCHIANTI [3]	23.449	6.014	11.661 to 35.237	<**0.01**
	AGE (years)	−0.406	0.341	−1.074 to 0.261	0.23
	GENDER (Female)	7.142	3.717	−0.144 to 14.428	0.05
	BMI (kg m^-2^)	−0.412	0.462	−1.317 to 0.493	0.37
	MMSE	0.093	0.894	−1.66 to 1.846	0.91
	GDS	−0.437	0.402	−1.225 to 0.351	0.27
